# Trends and research foci in immunoregulatory mechanisms of allergic rhinitis: a bibliometric analysis (2014-2024)

**DOI:** 10.3389/fimmu.2024.1443954

**Published:** 2024-09-24

**Authors:** Yandan Wang, Liangran Zhang, Baoyuan Shi, Junpeng Luo

**Affiliations:** ^1^ Department of Otolaryngology, Huaihe Hospital, Henan University, Kaifeng, China; ^2^ Translational Medical Center of Huaihe Hospital, Henan University, Kaifeng, China

**Keywords:** allergic rhinitis, immunoregulation, bibliometric analysis, NF kappa B, air pollution

## Abstract

**Background:**

This study aims to provide a comprehensive bibliometric analysis of research trends, hotspots, and future directions in the immunoregulatory mechanisms of allergic rhinitis (AR) from 2014 to 2024.

**Methods:**

Data were sourced from the Web of Science Core Collection (WoSCC), covering articles and reviews published between April 1, 2014, and March 31, 2024. The search terms included “Allergic Rhinitis,” “AR,” and related terms along with specific keywords related to immune cells and inflammatory mediators. Bibliometric tools such as CiteSpace, VOSviewer, and SCImago Graphica were used to analyze institutional cooperation networks, keyword co-occurrence, citation bursts, and research topic evolution. Microsoft Excel 2019 was employed to display annual publication trends.

**Results:**

A total of 2200 papers met the inclusion and exclusion criteria. The number of publications showed an upward trend over the past decade, with a significant peak in 2021. China (583 papers) and the United States (454 papers) were the major contributing countries. Imperial College London emerged as the leading institution. Key research frontiers identified include the roles of NF kappa B and air pollution in AR. Keyword burst analysis revealed emerging topics such as respiratory allergy and personalized treatment strategies. Notable limitations include the exclusive use of the WoSCC database and the restriction to English-language publications.

**Conclusion:**

The field of immunoregulatory mechanisms in allergic rhinitis has seen significant growth, with China and the United States leading the research. Future research should focus on developing personalized treatment plans and understanding the comprehensive impact of environmental factors. Continued interdisciplinary collaboration and international cooperation will be essential for advancing therapeutic strategies in AR.

## 1 Introduction

Allergic rhinitis (AR) is a prevalent chronic inflammatory condition affecting over 500 million individuals globally (1). The incidence of AR is notably higher in industrialized nations (2), with some countries reporting prevalence rates as high as 50% (1). Moreover, urbanization and industrialization have contributed to a rise in AR prevalence in developing countries, with the self-reported prevalence of pollen-induced allergic rhinitis (PiAR) in the grassland regions of Northern China reaches a remarkably high rate of 32.4% (3). The high incidence of AR imposes a substantial burden on overall health and has significant financial implications due to both direct and indirect costs associated with disease management. A recent study in the Netherlands estimated the total cost of AR to be €4,827 patient/year (4).

The pathogenesis of allergic rhinitis (AR) begins with the sensitization phase, where initial exposure to allergens such as pollen or dust mites induces the production of allergen-specific immunoglobulin E (IgE) (5). These IgE molecules bind to receptors on mast cells and basophils. Upon re-exposure to the same allergen, the IgE antibodies cross-link, triggering these cells to degranulate and release a cascade of inflammatory mediators, including histamines, leukotrienes, and cytokines. This process results in the characteristic symptoms of AR, such as nasal congestion, sneezing, itching, and rhinorrhea, and constitutes an immediate hypersensitivity reaction, also known as a Type I allergic reaction (6). The rising prevalence of AR and other allergic diseases is attributed to environmental changes and lifestyle factors that affect immune regulation, leading to an increased susceptibility to allergic sensitization and chronic inflammation (7). Understanding these mechanisms is crucial for developing effective therapeutic strategies (8, 9).

Given the increasing prevalence and impact of AR, studying its immunoregulatory mechanisms is of paramount importance. Understanding these mechanisms can lead to the development of more effective and personalized treatment strategies, thereby improving patient outcomes and reducing the economic burden associated with the disease (10–12).

Current treatments for AR, such as pharmacotherapy (13) and allergen-specific immunotherapy (AIT) (14), offer symptom relief to some extent, but they are not perfect (15, 16). These approaches face challenges including potential adverse reactions, variability in individual patient responses, issues with long-term treatment adherence, and economic burden (17, 18). Given these limitations, it is crucial to delve deeper into the immunoregulatory mechanisms of AR. Such research not only aids in developing new therapeutic strategies but also enables more precise personalized treatments, improves efficacy and safety, and helps prevent further disease progression.

To better understand the developments in this field, we have chosen to conduct a bibliometric analysis covering the period from 2014 to 2024. This ten-year analysis aims to capture and evaluate the evolution of research, revealing major trends and patterns in the field. This period has witnessed significant advancements in immunology and allergy research, allowing us to comprehensively outline the trajectory of research developments and shifts in focus within the scientific community. To the best of our knowledge, this is the first study to utilize bibliometrics and visual analysis to investigate trends in immune regulation mechanisms of allergic diseases.

## 2 Methods

### 2.1 Data collection

**Figure 1:**
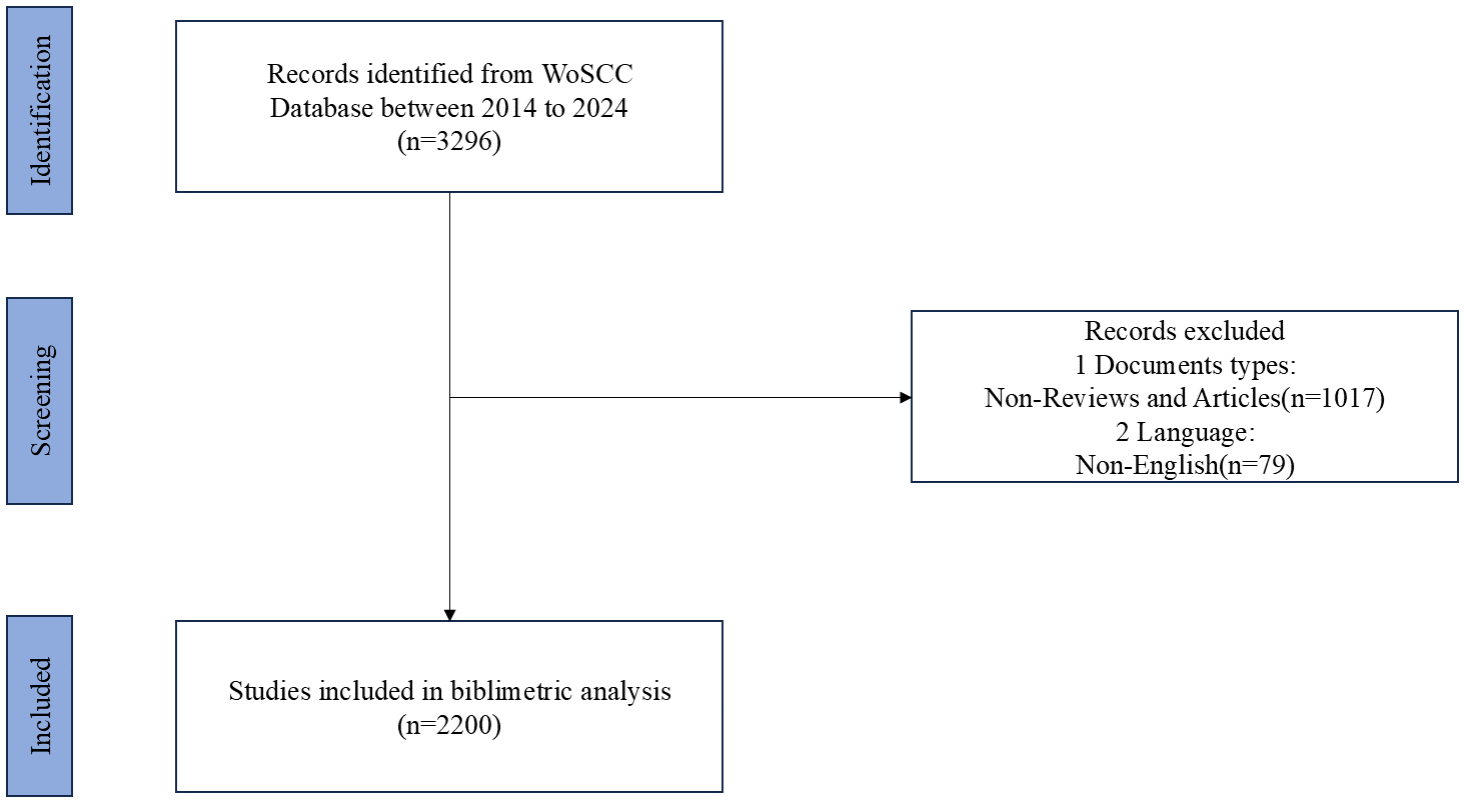
Flow diagram of literature selection and data screening.

Data for this study were sourced from the Web of Science Core Collection (WoSCC), a widely used citation database covering various academic research fields. To ensure data completeness and accuracy, the retrieval date was set as April 14, 2024. The search terms included “Allergic Rhinitis” or “AR” and related terms, along with specific keywords related to immune cells and inflammatory mediators. The search terms “(Allergic Rhinitis OR AR OR Rhinitis Allergic OR Allergic Rhinitides OR Rhinitides Allergic),” “(Immune Regulation OR Immune Cells OR Immunotherapy),” and “(Mast Cells OR Eosinophils OR T Lymphocytes OR Cytokines OR Interleukin OR Histamine OR IgE OR Allergens OR Inflammatory Mediators)” were used. The time range was set from April 1, 2014, to March 31, 2024, with language restricted to English and only including articles and reviews. **Figure 1** illustrates the literature search and screening process for this study.

### 2.2 Search strategy

The search strategy involved a comprehensive and systematic approach to identify relevant literature. Specific inclusion criteria were set to ensure the relevance of the selected articles: (1) studies focused on allergic rhinitis and its immunoregulatory mechanisms, (2) articles and reviews published in peer-reviewed journals, and (3) publications in English. Exclusion criteria included: (1) non-research articles such as editorials, letters, and conference abstracts, (2) studies not directly related to allergic rhinitis or its immunoregulatory mechanisms, and (3) duplicate records. This rigorous approach ensured that only pertinent studies were included in the analysis.

### 2.3 Data screening

After the initial data collection, literature screening was manually conducted by two independent researchers, Yandan Wang and Liangran Zhang, to ensure that relevant studies were selected based on predefined inclusion and exclusion criteria. Aside from the initial data collection phase, this process did not involve automated algorithms or specific screening programs. In the initial phase, bibliometric tools were used to retrieve relevant studies from databases. The manual screening process ensured the accuracy and consistency of the final dataset. Disagreements between researchers were resolved through discussion or consultation with domain experts such as Junpeng Luo and Baoyuan Shi. This dual screening approach effectively enhanced the accuracy and reliability of the data.

### 2.4 Data analysis

Bibliographic records were exported and saved in both “Full Record and Cited Reference” and “Plain Text” formats. To conduct an in-depth analysis of the research status and trends in the immunoregulatory mechanisms of allergic rhinitis, multiple bibliometric tools were utilized. These data were imported into CiteSpace, VOSviewer, and SCImago Graphica for further analysis. Additionally, journal categories and quartiles of Impact Factors (IF) were obtained from the 2023 edition of the Journal Citation Reports (JCR).

CiteSpace is a Java-based bibliometric tool used to reveal the knowledge structure, research hotspots, and trends in scientific fields. It provides standardized algorithms for clustering and trend analysis through the LLR algorithm and Kleinberg’s burst detection algorithm (19). CiteSpace can visualize networks of co-citations, keywords, and institutions, providing insights into the evolution and key areas of research.

VOSviewer constructs bibliometric networks based on data such as bibliographic coupling using co-occurrence matrices to visualize node similarity through distance (20). In this research, VOSviewer was used to generate keyword co-occurrence and author cooperation network graphs (21). Parameter settings included the “full counting” method, a minimum of five papers for authors, and a minimum co-occurrence threshold of 20 occurrences for keywords. VOSviewer’s visualization capabilities enable the identification of clusters of related items and trends over time.

SCImago Graphica is a comprehensive scientific analysis tool that provides detailed visualization capabilities for cooperation analysis at the national and institutional levels, integrated with SCImago Journal & Country Rank for macro-analysis convenience (22). It was used to create networks of national/regional cooperation and journals employing the “full counting” method and GML format to swiftly display data, identify research trends and cooperation patterns, and highlight potential research frontiers. SCImago Graphica’s ability to visualize and analyze geographical and institutional data offers unique insights into the collaboration patterns and impact of different regions and institutions.

Microsoft Excel 2019 was used to display the number of publications each year, offering a straightforward method for tracking publication trends over time (23).

## 3 Results

### 3.1 Publication trend analysis

**Figure 2:**
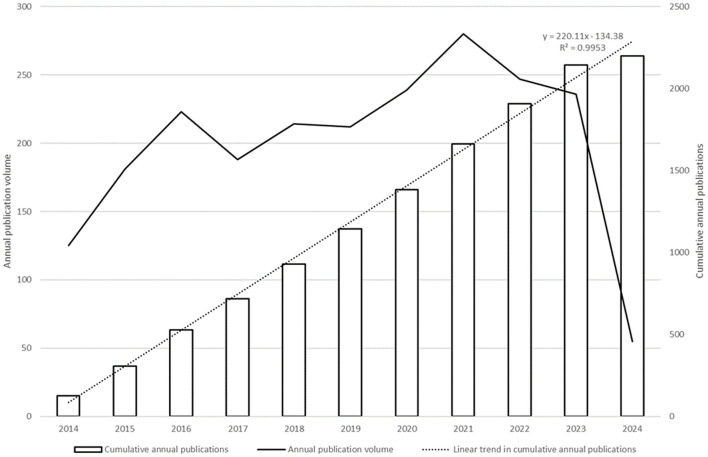
Statistics of Publications in the Relevant Field Worldwide Over the Past Decade. Annual Publication Volume: This line represents the number of publications made each year over the past decade. Cumulative Annual Publications: This line shows the cumulative total of publications up to and including each year.

From 2014 to 2024, a total of 2200 papers meeting the inclusion and exclusion criteria were identified. Despite slight fluctuations in certain years, the overall number of publications showed an upward trend. Particularly in 2021, global interest in the role of immune regulation mechanisms in allergic diseases peaked, with a total of 1327 related publications in the past five years, accounting for half of the entire research data. **Figure 2** illustrates the annual publication trend during this period.

### 3.2 Contributions from countries and institutions

**Figure 3:**
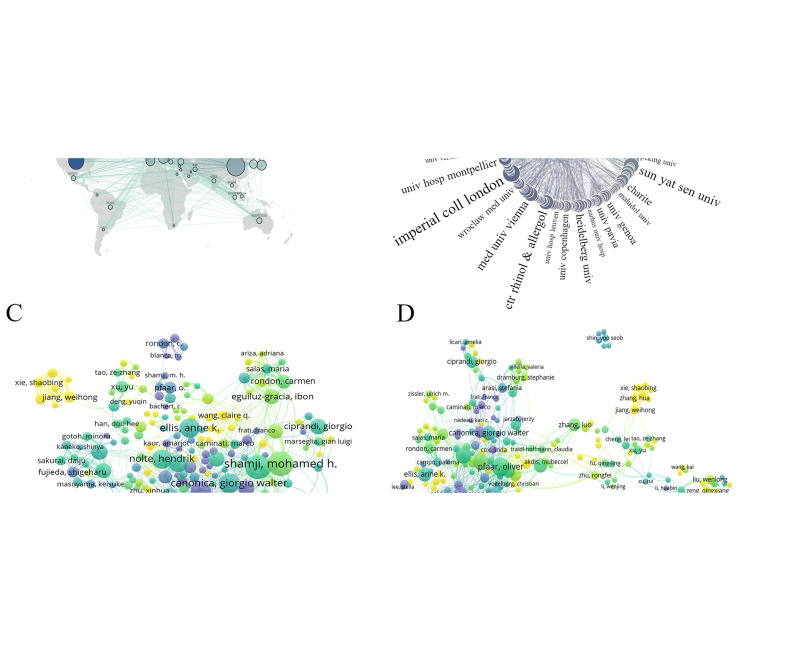
Global research output and collaboration in immunoregulatory mechanisms of allergic rhinitis. **(A)** Global distribution of publications and citations in immunoregulatory mechanisms of allergic rhinitis. **(B)** Institutional collaboration network. **(C)** Collaboration network of all authors with a production of ≥5 papers. **(D)** Collaboration network of the most prolific authors.

A total of 89 countries/regions and 420 institutions participated in these studies. **Figure 3A** illustrates the global distribution of research output and citations in the field of immunoregulatory mechanisms of allergic rhinitis. The size of the circles represents the number of publications, with larger circles indicating higher publication output. The color gradient of the circles indicates the number of citations, with darker colors representing higher citation counts. The map highlights significant contributions from China, the USA, and several European countries, showcasing their central role in global research collaboration. China (583 papers, 26.5%) and the United States (454 papers, 20.6%) were the major contributing countries, with other productive countries including Germany (257 papers, 11.7%), Italy (221 papers, 10.1%), and the United Kingdom (193 papers, 8.8%). Centrality score is an important indicator for quantitatively evaluating the importance of nodes in the network. In terms of centrality score, Norway (0.11), Ukraine (0.07), Australia (0.06), and Mexico (0.06) ranked at the top, indicating the significant role of these countries in international cooperation (**Table 1**).

**Table 1:** Ranking of the top 10 countries from 2014 to 2024.

Rank	Countries	Number of publications	Rank	Countries	Number of publications	Centrality
1	CHINA	583	1	NORWAY	13	0.11
2	USA	454	2	UKRAINE	9	0.07
3	GERMANY	257	3	AUSTRALIA	61	0.06
4	ITALY	221	4	MEXICO	45	0.06
5	ENGLAND	193	5	CHINA	583	0.05
6	JAPAN	166	6	GERMANY	257	0.05
7	SPAIN	160	7	ENGLAND	193	0.05
8	FRANCE	135	8	SWITZERLAND	77	0.04
9	SOUTH KOREA	117	9	TURKEY	68	0.04
10	POLAND	104	10	IRAN	36	0.04

**Figure 3B** displays the collaboration between institutions in the field of immunoregulatory mechanisms of allergic rhinitis. The size of the circles represents the number of documents produced by each institution, while the lines connecting the circles indicate collaboration between institutions. Imperial College London, Guangzhou Medical University, and Center for Rhinology are shown as major hubs with extensive collaborative networks, indicating their significant roles in advancing research through partnerships. Imperial College London published 72 papers, making it the most contributing institution (**Table 2**). This was followed by Guangzhou Medical University (63 papers), Center for Rhinology and Allergology (55 papers), Capital Medical University (43 papers), and Sun Yat-Sen University (41 papers). In the collaborative network (**Figure 3B**), frequent cross-regional collaborations among many institutions were observed. The more connections with other institutions, the more collaborations are evident.

**Table 2:** Ranking of the top 10 institutions from 2014 to 2024.

Rank	Institutions	Article Counts	Rank	Institutions	Article Counts	Centrality
1	Imperial Coll London	72	1	Johns Hopkins Univ	25	0.1
2	Guangzhou Med Univ	63	2	Ctr Rhinol & Allergol	55	0.08
3	Ctr Rhinol & Allergol	55	3	Erasmus MC	20	0.08
4	Capital Med Univ	43	4	Ajou Univ	12	0.07
5	Sun Yat Sen Univ	41	5	Univ Hosp Montpellier	36	0.06
6	Med Univ Vienna	39	6	Charite	30	0.06
7	Philipps Univ Marburg	38	7	Univ Genoa	29	0.06
8	Univ Zurich	37	8	Nippon Med Sch	24	0.06
9	Univ Hosp Montpellier	36	9	Beijing Inst Otolaryngol	22	0.06
10	Karolinska Inst	35	10	Merck & Co Inc	15	0.06

### 3.3 Authors and co-cited authors

A total of 10,309 authors contributed to this field, with a threshold of at least five published papers used to create the author collaboration network graph (**Figure 3C**). Oliver Pfaar from the Rhinology and Allergology Center in Germany was the most prolific scholar with 54 published papers and the highest number of collaborations in research, demonstrating his extensive academic influence and collaborative spirit (**Figure 3D**). This was followed by Stephen R. Durham (34 papers) and Mohamed H. Shamji (33 papers) from Imperial College London in the UK, and Pascal Demoly (33 papers). In terms of citation counts, Oliver Pfaar’s research was cited 2213 times, while Cezmi A. Akdis and Stephen R. Durham were cited 2102 and 1700 times, respectively (**Tables 3**, **4**).

**Table 3:** Top 10 Authors by number of publications from 2014 to 2024.

Rank	Authors	Article Counts	Cited Counts
1	Pfaar, Oliver	54	2213
2	Durham, Stephen R	34	1700
3	Shamji, Mohamed H	33	1527
4	Demoly, Pascal	33	1258
5	Klimek, Ludger	30	610
6	Akdis, Cezmi A	29	2102
7	Canonica, Giorgio Walter	26	652
8	Zhang, Luo	25	908
9	Bousquet, Jean	25	724
10	Ellis, Anne K	23	1149

**Table 4:** Top 10 Authors by centrality ranking from 2014 to 2024.

Rank	Authors	Article Counts	Centrality
1	Pfaar, Oliver	54	0.08
2	Ansotegui, Ignacio J	11	0.06
3	Canonica, Giorgio Walter	26	0.04
4	Calderon, Moises A	23	0.04
5	Bachert, Claus	21	0.04
6	Durham, Stephen R	34	0.03
7	Demoly, Pascal	33	0.03
8	Agache, Ioana	19	0.03
9	Cingi, Cemal	9	0.03
10	Shamji, Mohamed H	33	0.02

### 3.4 Journals and co-cited journals

Among the selected 2200 papers, they were published across 522 academic journals. The top 10 most productive journals collectively published 584 papers, accounting for 26.55% of the total (**Table 5**). Among them, “Allergy” (115 papers) and the “Journal of Allergy and Clinical Immunology” (82 papers) stand out as the most influential journals, cited 12,327 and 17,721 times, respectively, underscoring their pivotal role in allergic disease research.

**Table 5:** Ranking of the top 10 most productive journals from 2014 to 2024.

Journal	Counts	IF	JCR	Cited Journal	Cited Counts	IF	JCR
allergy	115	12	Q1	journal of allergy and clinical immunology	17721	14.2	Q1
journal of allergy and clinical immunology	82	14	Q1	allergy	12327	12.4	Q1
international archives of allergy and immunology	57	2.8	Q3	clinical and experimental allergy	4721	6.1	Q1
clinical and experimental allergy	55	6.1	Q1	Annals of Allergy, Asthma & Immunology	3373	5.9	Q2
frontiers in immunology	53	7.3	Q1	The Journal of Immunology	2811	4.4	Q2
international forum of allergy & rhinology	47	6.4	Q1	International Archives of Allergy and Immunology	2153	2.8	Q3
journal of allergy and clinical immunology-in practice	47	9.4	Q1	Journal of Allergy and Clinical Immunology-In Practice	1557	9.4	Q1
clinical and translational allergy	46	4.4	Q2	Pediatric allergy and immunology	1533	4.4	Q1
allergy and asthma proceedings	41	2.8	Q3	The New England Journal of Medicine	1304	158.5	Q1
international immunopharmacology	41	5.6	Q1	Plos one	1180	3.7	Q2

**Figure 4:**
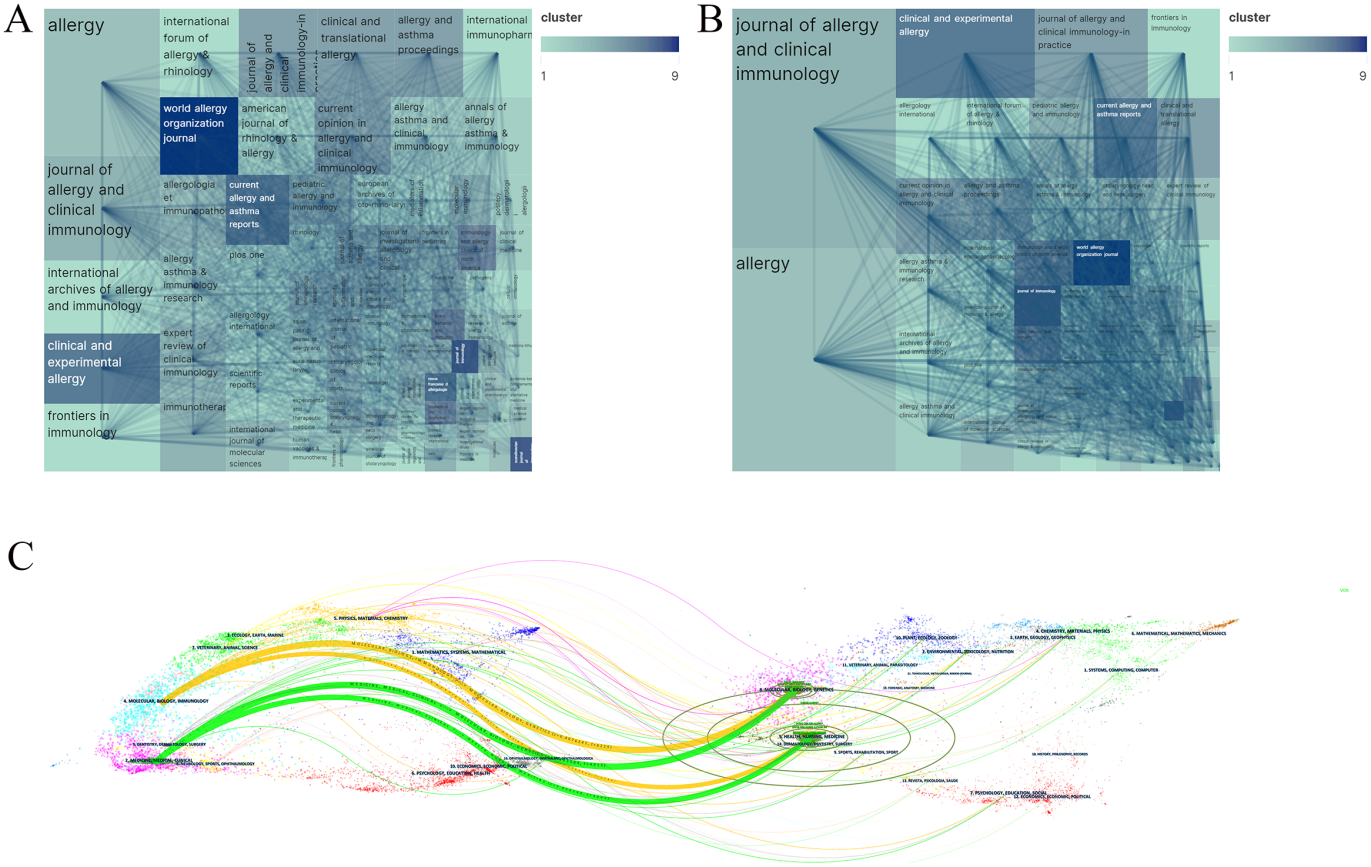
Journal output and collaboration network. **(A)** Explain the output and collaboration status of academic journals. **(B)** Analyze journal citations. The threshold for selection is set as journals with a cumulative citation count ≥200. **(C)** A dual-map overlay analysis of journals.

From **Table 5**, it is observed that among the top 10 journals with the most citations, all have been cited over 1000 times. Specifically, two journals have exceeded 10,000 citations each: the “Journal of Allergy and Clinical Immunology” (17,721 citations) and “Allergy” (12,327 citations). The journal with the highest impact factor is the “New England Journal of Medicine” (IF = 158.5), followed by the “Journal of Allergy and Clinical Immunology” (IF = 14.2). Selecting journals with a minimum citation count of 200, a co-citation network was constructed as depicted in **Figures 4A, B**.

The overlay of dual maps of journals illustrates the thematic distribution of relationships between journals (**Figure 4C**). On the left side of the map are the citing journals, while on the right are the cited journals. Within the map, four main citation paths reveal the reciprocal influence and mutual progress between molecular biology, immunology, and clinical medicine. They indicate that without advancements in the knowledge structures of molecular biology and immunology, the development of allergy-related clinical medicine would be challenging. Conversely, without the validation and feedback from clinical medicine, the fields of molecular biology and immunology would struggle to move forward. This interdisciplinary field primarily encompasses disciplines such as medicine, molecular biology, and immunology.

### 3.5 Keywords co-occurrence and burst analysis

**Figure 5:**
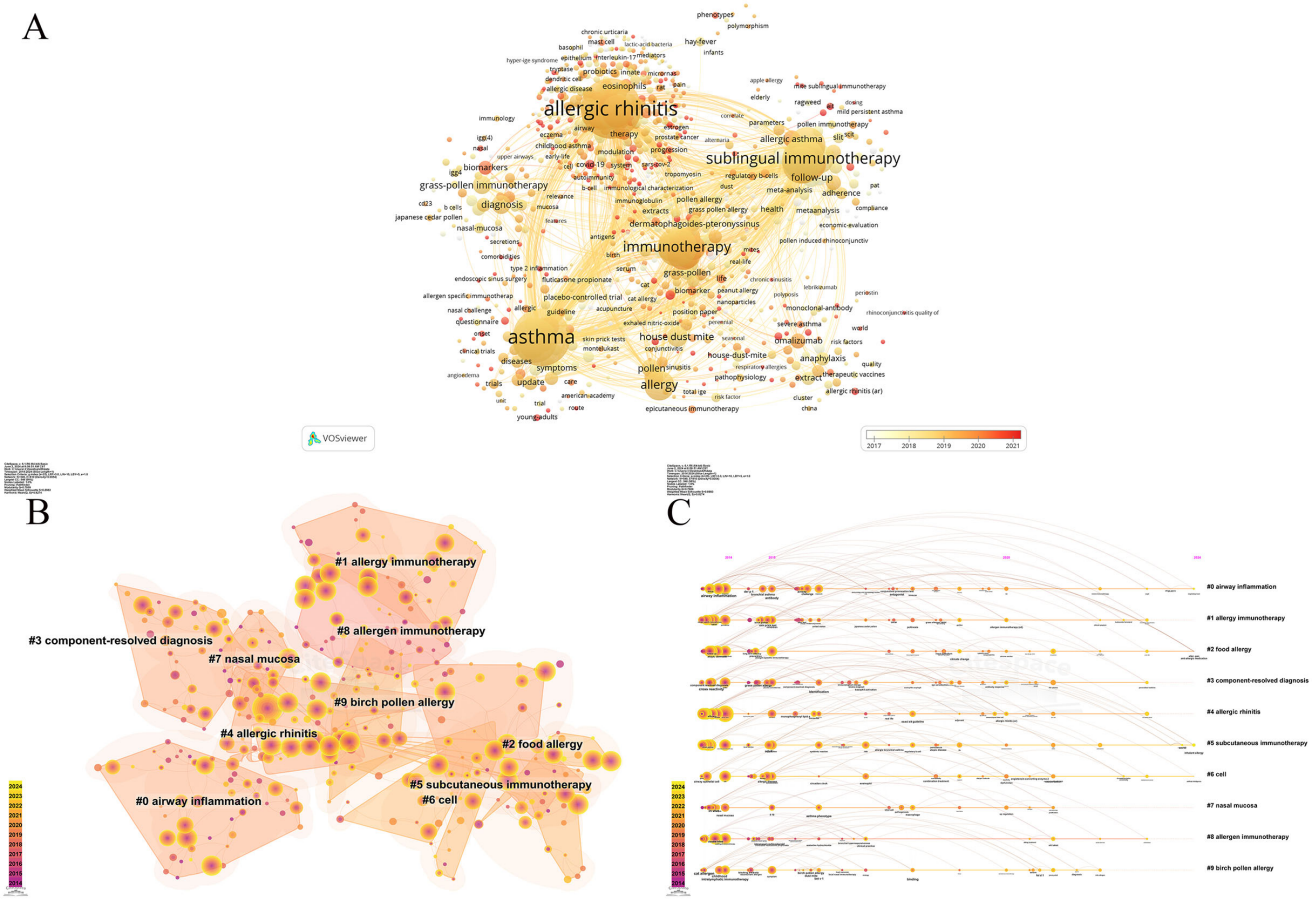
Keyword analysis and research clusters in allergic rhinitis immunoregulation. **(A)** keyword co-occurrence analysis. **(B)** Keyword clustering analysis. **(C)** Keyword co-occurrence timeline.

The co-occurrence analysis of keywords reveals a total of 7064 extracted keywords, with 184 keywords appearing more than 20 times. The **Figure 5A** illustrates the keyword co-occurrence analysis, revealing the evolving focus of research on the immunoregulatory mechanisms of allergic rhinitis from 2014 to 2024. Major topics such as “asthma” (825 occurrences), “allergic rhinitis” (793 occurrences), “sublingual immunotherapy” (448 occurrences), “rhinitis” (426 occurrences), and “immunotherapy” (393 occurrences) have consistently garnered significant interest. In the earlier years, research emphasized respiratory allergy, randomized controlled trials, prevalence, efficacy, subcutaneous immunotherapy, expression, and response. Since 2018, there has been a notable shift towards NF-kappa B, pathways, macrophages, proteins, regulatory B cells, allergen challenges, and air pollution (**Figure 5A**).

Clustering analysis of keywords helps identify the structural framework of a relevant research field. Each cluster represents a distinct research theme, with the size of the nodes indicating the frequency of keyword occurrences. By selecting the top 10 clusters based on the strength of keyword co-occurrence links, different-colored circles represent the appearance of years (**Figure 5B**). A timeline view based on the co-occurrence of keywords aids in visualizing phased research hotspots and directions over time (**Figure 5C**). Over the past decade, research directions have focused on specific immunotherapy, intralymphatic immunotherapy, sublingual and subcutaneous immunotherapy, regulatory T cells, chronic rhinosinusitis, immune response, immune system, and risk factors. The depth of color in this area indicates that keywords are distributed sequentially over time.

**Figure 6:**
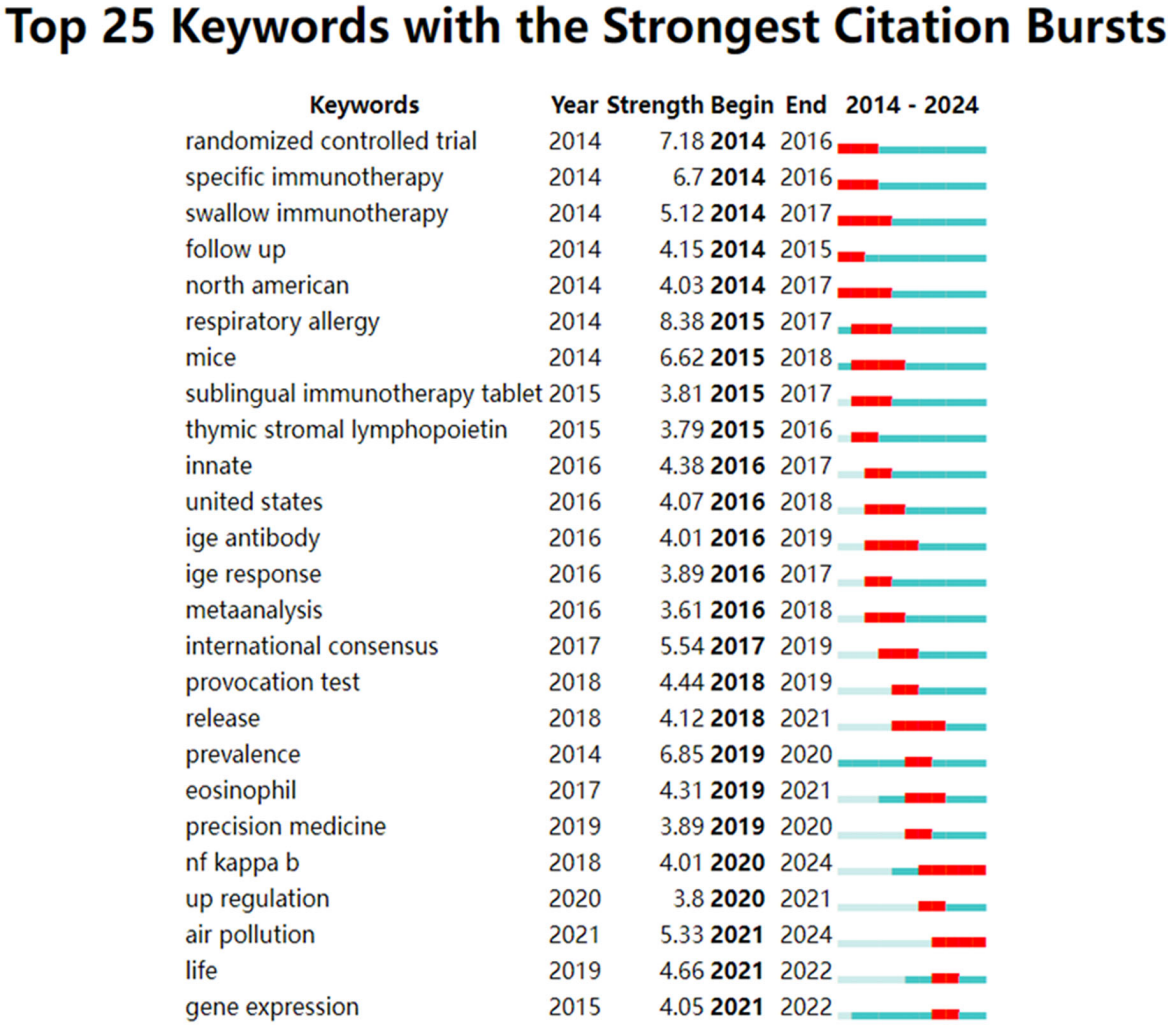
The top 25 keywords with the strongest citation bursts related to the immunoregulatory mechanism of allergic rhinitis.

Keyword bursts elucidate trends in the development of this field. **Figure 6** displays the top 25 keywords with the strongest bursts. From 2014 to 2024, “respiratory allergy” had the highest burst strength (8.38), followed by “randomized controlled trial” (7.18) and “prevalence” (6.85). Additionally, “NF kappa B” and “prevalence” maintained burst strength until the publication year of 2024, reflecting the latest research trends.

### 3.6 Cited references and citation burst analysis

Cited references refer to documents cited by one or more publications simultaneously, representing the knowledge base of a specific field. The article by Bousquet et al., published in “Allergy” in 2008, has the highest number of citations (397), followed by the works of Cox et al., published in the “Journal of Allergy and Clinical Immunology” in 2011 (238 citations), and Brozek et al., published in the same journal in 2017 (178 citations) (**Table 6**).

**Table 6:** Top 10 most co-cited references from 2014 to 2024.

Cited References	Citation Counts
bousquet j, 2008, allergy, v63, p8, doi 10.1111/j.1398-9995.2007.01620.x	397
cox l, 2011, j allergy clin immun, v127, p0, doi 10.1016/j.jaci.2010.09.034	238
brozek jl, 2017, j allergy clin immun, v140, p950, doi 10.1016/j.jaci.2017.03.050	178
roberts g, 2018, allergy, v73, p765, doi 10.1111/all.13317	177
jacobsen l, 2007, allergy, v62, p943, doi 10.1111/j.1398-9995.2007.01451.x	176
brozek jl, 2010, j allergy clin immun, v126, p466, doi 10.1016/j.jaci.2010.06.047	169
durham sr, 2012, j allergy clin immun, v129, p717, doi 10.1016/j.jaci.2011.12.973	145
canonica gw, 2014, world allergy organ, v7, p0, doi 10.1186/1939-4551-7-6	143
durham sr, 1999, new engl j med, v341, p468, doi 10.1056/nejm199908123410702	142
pfaar o, 2014, allergy, v69, p854, doi 10.1111/all.12383	134

**Figure 7:**
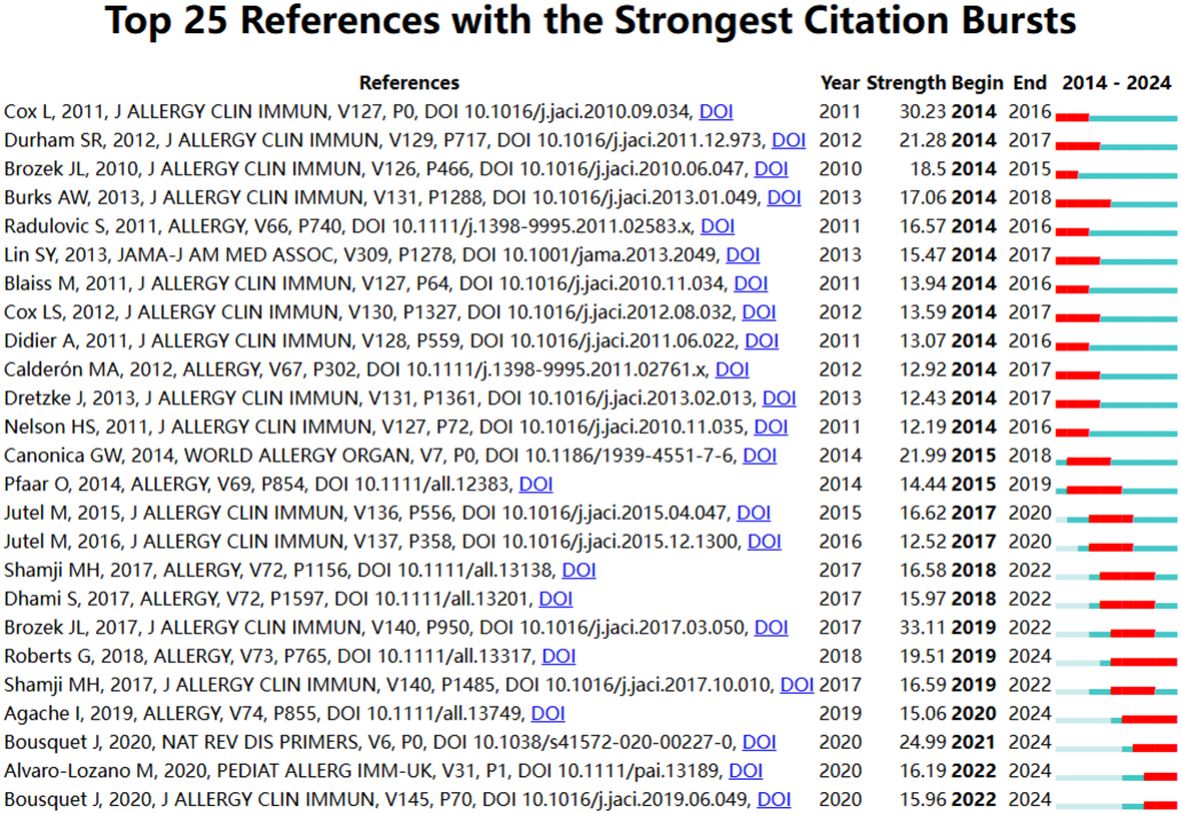
The top 25 cited articles with the strongest citation bursts related to the immunoregulatory mechanism of allergic rhinitis.

Detecting citation bursts in referenced literature can indicate the evolution of hotspots over time and future trends in a particular field. **Figure 7** displays the top 25 referenced documents with the strongest citation bursts. Among them, the 2017 publication by Brozek JL et al. in “J Allergy Clin Immun” titled “Allergic Rhinitis and its Impact on Asthma (ARIA) guidelines—2016 revision” exhibits the strongest citation burst. Additionally, papers including “Next-generation Allergic Rhinitis and Its Impact on Asthma (ARIA) guidelines for allergic rhinitis based on Grading of Recommendations Assessment Development and Evaluation (GRADE) and real-world evidence,” “Allergen Immunotherapy in Children User’s Guide,” “Allergic rhinitis,” and two sets of “EAACI Guidelines on Allergen Immunotherapy” have sustained citation bursts until 2024.

**Figure 8:**
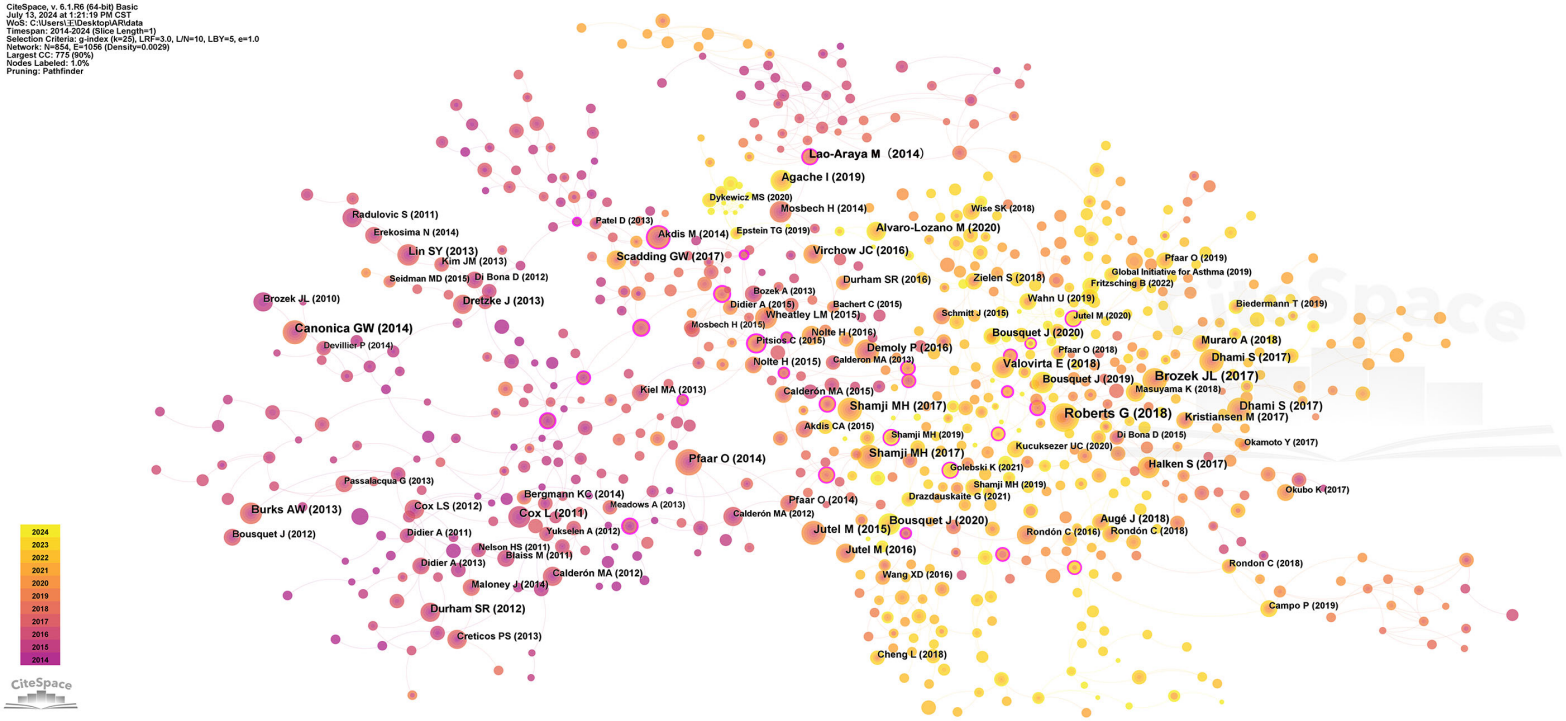
Co-citation analysis of references.

**Figure 8** illustrates the co-citation network of references in the research on immunoregulatory mechanisms of allergic rhinitis from 2014 to 2024. This cited reference network consists of 854 nodes and 1056 links. Each node represents a cited reference, and the size of the node indicates the number of citations. The lines connecting nodes represent co-citation relationships. Prominent references, such as those by Canonica GW (2014), Brozek JL (2010, 2017), and Pfaar O (2014), are highlighted due to their high citation counts and central roles in the network. The color gradient from purple (2014) to yellow (2024) shows the timeline, indicating how the focus of citations has shifted over time. This analysis helps identify influential studies and emerging trends in the field.

**Figure 9:**
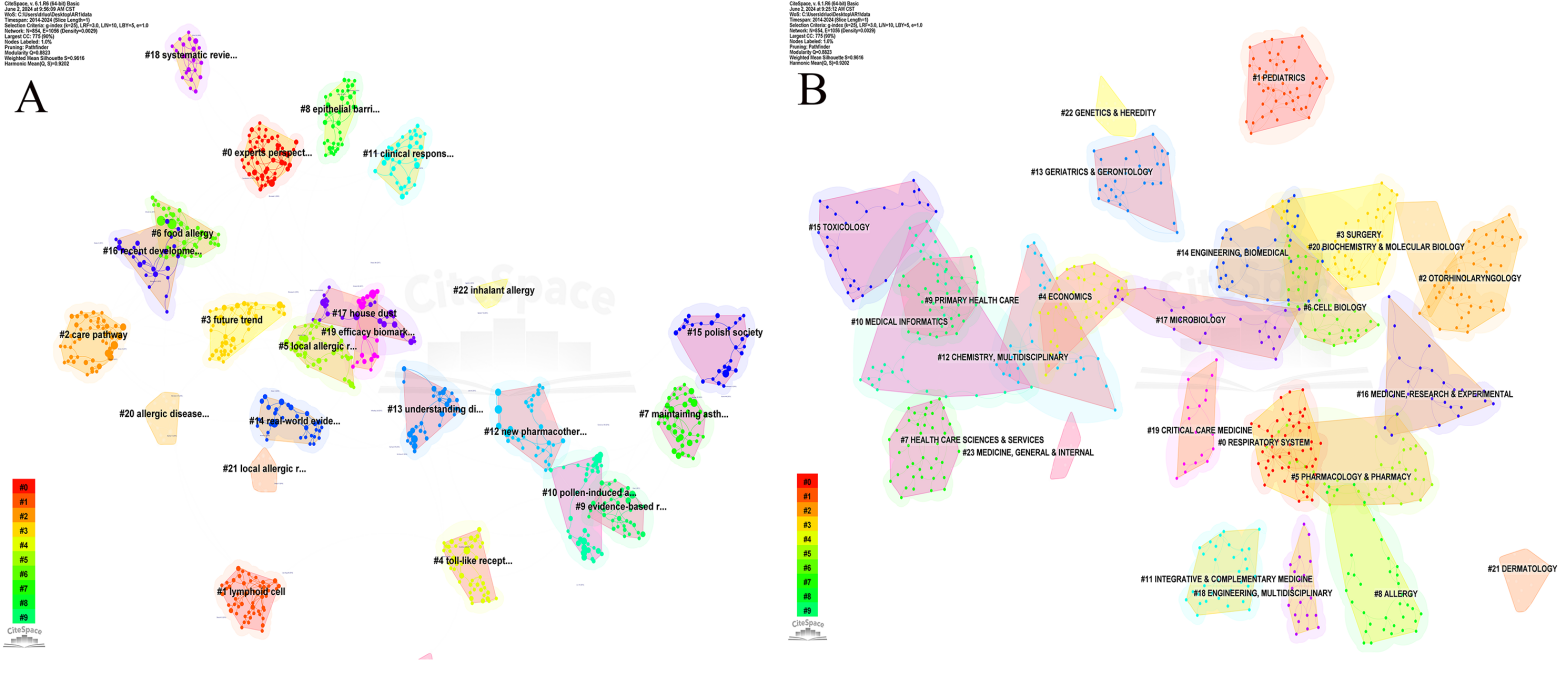
Clustering analysis of co-cited references. **(A)** Clustering analysis of research directions. **(B)** Clustering analysis of research themes categories.

In-depth analysis reveals that these documents represent 24 major research directions in the field, with the most significant ones being “experts perspective” (#0), “lymphoid cell” (#1), “care pathway” (#2), “future trend” (#3), “toll-like receptor” (#4), “local allergic rhinitis” (#5), and “food allergy” (#6). The recent ongoing directions include “experts perspective” (#0) and “inhalant allergy” (#22) (**Figure 9A**). The main research areas are concentrated in “respiratory system” (#0), “pediatrics” (#1), “otorhinolaryngology” (#2), “surgery” (#3), “economics” (#4), “pharmacology & pharmacy” (#5), and “cell biology” (#6). Currently, “respiratory system” (#0) and “genetics & heredity” (#22) remain important research areas (**Figure 9B**).

## 4 Discussion

### 4.1 General information

The publication trends in allergic rhinitis (AR) and immunoregulation over the past decade demonstrate a marked increase in research output, reflecting a growing global awareness of allergic diseases. Countries such as China and the United States are leading contributors, likely due to robust research funding and strategic prioritization of allergic disease studies. This increased focus has been crucial in addressing the rising prevalence of AR and other allergic conditions, which are influenced by environmental changes and lifestyle factors.

High-impact institutions, including Imperial College London and Guangzhou Medical University, have emerged as key players in this field. These institutions not only produce a significant volume of research but also influence the direction of studies through collaborative networks and partnerships with other leading entities. The concentration of prolific authors in these institutions further accentuates their role in advancing the field, as these researchers frequently collaborate across borders, enhancing the quality and reach of the research.

Advanced bibliometric tools such as VOSviewer have provided valuable insights into these collaborative networks, highlighting the interconnected nature of the research community. The mapping of co-authorship and institutional collaborations reveals a dense web of interactions that facilitate knowledge sharing and innovation. This network structure is particularly important in identifying and developing emerging research areas, such as personalized medicine in AR, which aims to tailor treatments based on individual patient profiles.

Overall, these findings underscore the importance of continued investment in research infrastructure and international cooperation. As allergic diseases continue to pose significant public health challenges, the research community must leverage these collaborative frameworks to explore new therapeutic avenues and improve patient outcomes. The future of AR research lies in fostering interdisciplinary collaboration and integrating new technologies to deepen our understanding of immunoregulatory mechanisms.

### 4.2 Implications for clinical practice and future research

These publications provide guidance for future research on AR, emphasizing the development of personalized treatment strategies, focusing on patient quality of life, enhancing the representativeness of clinical trials, and strengthening the generalizability of research. Research should continue to investigate the efficacy of AIT in treating allergic asthma and use the GRADE approach to develop treatment recommendations. Future research priorities also include exploring the impact of environmental exposure and genetic background on AR and the application of mobile technologies in personalized treatment.

For AIT treatment of allergic diseases in children, research should focus on clinical outcomes, immune mechanisms, indications, and routes of administration. Updates to the ARIA guidelines should consider the GRADE guidelines and real-world evidence to optimize drug treatment strategies and explore the application of molecular diagnostics and digital health systems in AIT management.

A keyword co-occurrence analysis reveals that the focus of research on the immunoregulatory mechanisms of allergic rhinitis has been gradually shifting. From 2014 to 2024, topics such as allergic rhinitis, asthma, sublingual immunotherapy, immunotherapy, and allergy have consistently been major points of interest for researchers. In the early part of this period, there was more emphasis on respiratory allergy, randomized controlled trials, prevalence, efficacy, subcutaneous immunotherapy, expression, and response. Since 2018, there has been increased attention on NF-kappa B, pathways, macrophages, proteins, regulatory B cells, allergen challenges, and air pollution. NF-kappa B and air pollution, in particular, continue to influence current research, reflecting the latest trends in the field.

NF kappa B is an important protein complex that regulates inflammation, immune responses, cell proliferation, and apoptosis within cells (24). It is expressed in various cells and is crucial for maintaining cellular stability and responding to external stimuli (25). NF kappa B is inactive when bound to the inhibitory protein Ikappa B in the cytoplasm (26). Upon receiving inflammatory or stress signals, Ikappa B kinase activates and phosphorylates Ikappa B, leading to its ubiquitination and degradation, thereby releasing NF kappa B. NF kappa B then enters the nucleus, activating the transcription of genes encoding inflammatory mediators, which play a role in immunity and inflammation (27). NF kappa B is also a significant target in the treatment of autoimmune diseases (28), chronic inflammation (29), and cancer (30).

In AR, NF kappa B exacerbates symptoms by regulating inflammation and activating immune cells (31). It promotes the Th2 immune response and the production of inflammatory cytokines (32), making it a potential therapeutic target. Drugs such as corticosteroids can alleviate symptoms by inhibiting NF kappa B activity (33), offering new strategies for controlling AR inflammation. A deeper understanding of the role of NF kappa B in AR is crucial for developing future treatment strategies.

Air pollution is closely related to AR because harmful substances like particulate matter, nitrogen dioxide, and ozone can exacerbate symptoms, trigger inflammation, and cause immune imbalance, increasing sensitivity to allergens (34). During pollen season, the interaction between air pollutants and pollen can further worsen AR symptoms (35). Long-term exposure to polluted environments may also increase the risk of chronic inflammation and oxidative stress (36, 37), which are closely related to the development and exacerbation of AR. Effective mitigation of AR symptoms and improvement of treatment outcomes require reducing exposure to air pollution, necessitating joint efforts in public health policies and individual lifestyle changes.

Current research focuses on the specific mechanisms by which air pollution affects AR, including analyzing how pollutants activate inflammatory and immune responses through molecular pathways (38). Epidemiological and Geographic Information System (GIS) technology analyses examine how pollutants trigger inflammation and immune responses and their connection to AR incidence (39). Concurrently, clinical trials and intervention studies evaluate the effectiveness of reducing pollution exposure or medical interventions in improving AR symptoms (40). Personalized medical strategies are being explored based on individual genetics (41), lifestyle (9, 41, 42), and environmental exposure (43). Research on children with AR is also being strengthened to address their sensitivity to air pollutants (44). New technologies, such as remote sensing monitoring (45), the Internet of Things (IoT) (46), and mobile health technologies (47), provide new avenues for real-time air quality monitoring and assessing its impact on AR symptoms. Studies on policy and public health interventions are underway to reduce air pollution, improve the health of AR patients, and encourage interdisciplinary collaboration, combining environmental science (48), medicine (49), biology (50), epidemiology (51), and data science (52) to fully understand the relationship between air pollution and AR and develop more effective prevention and treatment strategies (35, 53).

Bibliometric analysis results provide guidance for clinical practice and future research in the AR field, revealing new therapeutic targets, such as the roles of NF kappa B and air pollution. Future research will be dedicated to developing personalized treatment plans (including genetic, environmental, and lifestyle factors) for precision medicine. Clinicians can benefit from understanding these emerging trends, incorporating the latest research into their treatment strategies, potentially improving patient outcomes. Future research should also focus on exploring different immune regulatory pathways in allergic diseases to find more effective treatment strategies. In the future, this field will integrate more secondary disciplines to study mechanisms related to immune regulation more precisely, ensuring personalized healthcare. Interdisciplinary collaboration and international cooperation will be key drivers of scientific progress in this field.

### 4.3 Limitations

Despite the comprehensive approach of this bibliometric analysis, several limitations should be noted. Firstly, the literature was exclusively extracted from the WoSCC database, which may result in an incomplete and biased inclusion of studies. Future studies should consider including multiple databases, such as PubMed, Scopus, and Google Scholar, to enhance the comprehensiveness of the data. Secondly, only English articles and reviews were included, which may exclude relevant research published in other languages and potentially lead to language bias.

Additionally, despite standardized procedures, certain biases may persist due to differences in keyword phrasing, identical author names, and continuous updates to the WoSCC database. Addressing these limitations in future research will enhance the accuracy and reliability of findings.

## 5 Conclusion

This bibliometric analysis highlights significant growth in research on the immunoregulatory mechanisms of allergic rhinitis over the past decade, with China and the United States as leading contributors and major research frontiers such as NF kappa B and air pollution. Developing personalized treatment plans and understanding environmental impacts are essential for advancing therapies. Interdisciplinary collaboration and international cooperation are crucial for further progress. These insights guide future research, emphasizing the importance of continued global and interdisciplinary efforts in enhancing allergic rhinitis treatment.

## Data Availability

The raw data supporting the conclusions of this article will be made available by the authors, without undue reservation.
